# Community Acquired Bacteremia in Young Children from Central Nigeria- A Pilot Study

**DOI:** 10.1186/1471-2334-11-137

**Published:** 2011-05-19

**Authors:** Stephen Obaro, Lovett Lawson, Uduak Essen, Khalid Ibrahim, Kevin Brooks, Adekunle Otuneye, Denis Shetima, Patience Ahmed, Theresa Ajose, Michael Olugbile, David Idiong, Damola Ogundeji, Comfort Ochigbo, Grace Olanipekun, Walid Khalife, Richard Adegbola

**Affiliations:** 1 Michigan State University, East Lansing 48824, Michigan, USA; 2 Visiting Consultant Paediatrician, National Hospital, Abuja, Nigeria; 3 Chief Medical Director, Zankli Medical Center, Abuja, Nigeria; 4 Medical Research Council Laboratories, Fajara, The Gambia

## Abstract

**Background:**

Reports of the etiology of bacteremia in children from Nigeria are sparse and have been confounded by wide spread non-prescription antibiotic use and suboptimal laboratory culture techniques. We aimed to determine causative agents and underlying predisposing conditions of bacteremia in Nigerian children using data arising during the introduction of an automated blood culture system accessed by 7 hospitals and clinics in the Abuja area.

**Methods:**

Between September 2008 and November 2009, we enrolled children with clinically suspected bacteremia at rural and urban clinical facilities in Abuja or within the Federal Capital Territory of Nigeria. Blood was cultured using an automated system with antibiotic removing device. We documented clinical features in all children and tested for prior antibiotic use in a random sample of sera from children from each site.

**Results:**

969 children aged 2 months-5 years were evaluated. Mean age was 21 ± 15.2 months. All children were not systematically screened but there were 59 (6%) children with established diagnosis of sickle cell disease and 42 (4.3%) with HIV infection. Overall, 212 (20.7%) had a positive blood culture but in only 105 (10.8%) were these considered to be clinically significant. Three agents, *Staphylococcus aureus *(20.9%), *Salmonella typhi *(20.9%) and Acinetobacter (12.3%) accounted for over half of the positive cultures. *Streptococcus pneumoniae and non-typhi Salmonellae *each accounted for 7.6%. Although not the leading cause of bacteremia, *Streptococcus pneumoniae *was the single leading cause of all deaths that occurred during hospitalization and after hospital discharge.

**Conclusion:**

*S. typhi *is a significant cause of vaccine-preventable morbidity while *S. pneumoniae *may be a leading cause of mortality in this setting. This observation contrasts with reports from most other African countries where non-typhi Salmonellae are predominant in young children. Expanded surveillance is required to confirm the preliminary observations from this pilot study to inform implementation of appropriate public health control measures.

## Background

Sub Saharan Africa contributes significantly to the global mortality of children aged less than 5 years with mortality rates of 100-250 per 1000 [1]. It is now well established that invasive bacteria disease is the leading cause of childhood mortality in sub-Saharan Africa and most of these infections are preventable by the use of vaccines which are already licensed and in routine use in most developed countries. Few health facilities in Africa have the microbiologic laboratories with capacity to identify invasive bacterial infection. Thus the clinical importance of bacterial infections and associated mortality is often not recognized with most febrile illnesses being attributed to malaria. Randomized controlled efficacy trials in Gambia [2,3]. and South Africa [4] and effectiveness studies in Malawi [5] uncovered substantial disease burden from *Haemophilus influenzae *type b (Hib) and *Streptococcus pneumoniae*, while population based surveillance studies in Kenya have demonstrated substantial disease caused by *S. pneumonia *[6]. While *S. pneumoniae *has been identified as the leading cause of bacteremia or invasive bacteria disease in The Gambia [7], Kenya [6], Mozambique [8], this was not the case in Ghana [9] and Malawi [10] where non-typhi Salmonella (NTS) predominated. Availability of national epidemiologic data will strengthen advocacy for the introduction of relevant vaccines.

Although Nigeria is the most populated country in the sub region and records high infant and under-5 mortality rates of 97 and 189 per 1000 respectively [11], data on the etiologic agents of invasive bacteria disease in children are sparse.

Primary health care system is poorly developed in the country and chemists, pharmacies, private clinics and hospitals are often the first port of call for parents/guardians of children seeking care for their children. Over-the-counter antibiotic use is very prevalent and this is likely to impact the outcome of any studies aimed at the determination of the causes of bacteria infection in children. It is possible that this healthcare seeking behavior may of itself modify the spectrum of prevalent bacteria pathogens and also the spectrum of clinical disease presentation. Previous reports of causes of bacteremia from different parts of Nigeria utilized conventional blood culture techniques with the use of human blood for the preparation of blood agar and suboptimal methods for the detection of fastidious organisms like *S.pneumoniae*. Limitations of this approach have been reviewed [12]. Against this background, we sought to introduce surveillance blood cultures using an automated system, providing access to the facility from 7 hospitals and clinics in the Abuja area. Using data arising from this pilot surveillance, we report the commonly identified causes of community-acquired bacteremia in children aged 2 months-5 years in an urban and semi-urban setting with reference to vaccine-preventable diseases.

## Methods

### Ethics Approval

This study was approved by the Federal Capital Territory of Nigeria Ethics Committee, Zankli Medical Center Ethics Committee, National Hospital Abuja Ethics Committee, Keffi Medical Center Ethics Committee, The Joint Gambia Government and MRC Ethics Committee, and Michigan State University Institutional Review Board.

### Setting

Nigeria is the most populated country in sub-Saharan Africa with a population of almost 150 million [13]. Abuja is the capital city of Nigeria, located in the Federal Capital Territory (FCT) which is the geographical centre of Nigeria. It has a land area of 8,000 square kilometers. It is bounded on the north by Kaduna State, the west by Niger State, the east and southeast by Nassarawa State and the southwest by Kogi State. Abuja is a "planned" city as it was mainly built in the 1980s and officially became Nigeria's capital in 1991, replacing the previous capital in Lagos. In 2006 the population was estimated at 1.7 million but may currently be about 5.7 million [13]. Abuja and the FCT have experienced a huge population growth; it has been reported that some areas around Abuja have been growing at an annual rate of 20 - 30%. The rapid spread of squatter settlements and shanty towns in and around the city limits is believed to be an important contributor to this rapid growth. The rainy season begins in April and ends in October. Within this period there is a brief interlude of harmattan, occasioned by the Northeast Trade Wind, with the main features of dust haze, intensified coldness and dryness. The annual total rainfall is in the range of 1,100 to 1,600 mm. The population is diverse, with increasing representation from the major ethnic groups of Hausa, Yoruba and Igbos following the development of the FCT and relocation of the federal capital.

There is perennial malaria transmission, mostly due to *Plasmodium falciparum *and HIV prevalence of 5.6% among pregnant women attending antenatal clinics.

#### National Immunization Program

The national expanded program on immunization calendar in Nigeria includes Bacillus Calmette Guerin vaccination, oral polio vaccine (OPV) and Hepatitis B at birth, OPV, diphtheria-tetanus whole cell pertussis (DPT) and Hepatitis B at 6 weeks, OPV and DPT 10 and 14 weeks, Hepatitis B and Measles at 9 months and Yellow Fever at between 9-12 months. National immunization coverage varies across the country but in the FCT has been about 85% for DTP3 [14]. Additional vaccines such as conjugate pneumococcal and *Haemophilus influenzae *type b are available at a few private clinics.

In 2008, Michigan State University in collaboration with the National Hospital Abuja established a research project to determine the etiologic agents of community-acquired bacteremic syndromes (sepsis, pneumonia, meningitis and bacteremia) in young Nigerian children aged 2 months -5 years. The enrollment sites for this Community-acquired Bacteremic Syndrome (CABSYNC) study include a number of hospitals within Abuja city and other rural settlements on the outskirt of Abuja, carefully selected to capture a mix of urban and rural population within easy reach of the diagnostic laboratories in Abuja city. Together, these facilities have an annual pediatric outpatient attendance of over 110,000 and 12,000 admissions and include the following;

##### a) The National Hospital, Abuja

The National Hospital of Abuja was originally designed to cater to the needs of women and children in Nigeria and the West African sub-region, with a view to reducing morbidity and mortality rates, and to carrying out extensive research into the particular causes of diseases in women and children in Africa. Phase 1 of the Hospital presently contains 200 beds, but the centre has facilities for a future expansion to 500 beds. The pediatric department admits about 90-100 patients per month and attends to 1300-1500 patients per month in the out-patient department. The facility provides postgraduate training in Pediatrics and has eight Attending Pediatricians and twenty five resident doctors.

##### b) University of Abuja Teaching Hospital, Gwagwalada

This is a district general hospital which has just recently been designated the teaching hospital for the University of Abuja. The hospital has a 300 bed capacity and serves a rural population from several villages and settlements on the outskirt of Abuja, within the Federal Capital Territory and also from the adjoining states. There are currently three Attending Pediatricians with 14 residents and with its new status; the university is in the process of employing more attending Pediatricians.

##### c) Nyanya District Hospital

This is a small district hospital located on the outskirts of Abuja and functions as a primary health care center with limited in-patient capacity. This facility is staffed by five generalist physicians, supported by nurses and midwives. Patients are generally referred to the National Hospital for specialist care. The facility has minimal laboratory support for urine microscopy and malaria blood films.

##### d) Zankli Medical Center

Zankli Medical Center (ZMC) is a private hospital based in Abuja. It has approximately 150 members on staff, 45 beds for in-patient admission, 17 full time doctors, including 9 consultants. In addition to the above there are four independent specialist clinics: Dental, Dermatology, Ophthalmology and ENT, which operate within the hospital premises. Constant power and water supplies are guaranteed by in-house generators to maintain 24-hour electricity and a borehole as a source of water. Communication is facilitated through broadband internet access and a network system within the hospital maintained by an in-house engineer. The Hospital is approved by the National Postgraduate Medical College as a tutelage centre for senior registrars in general practice and has recently embarked on the development of academic and research activities in collaboration with the Federal Ministry of Health of Nigeria and Liverpool School of Tropical Medicine, UK. The facility provides care for over 30,000 pediatric outpatients annually, with 1,255 admissions. The Research Laboratory has two senior laboratory scientists and ten other laboratory scientists working in the routine laboratory. The lab is equipped with two light microscopes, one fluorescent microscope, a Bactec™ MGIT machine for mycobacterial culture, and a Bactec™ 9050 for bacterial culture.

##### e) Garki General Hospital

Garki Hospital Abuja (GHA) is a private public hospital established in 1988. The hospital is located within less than 5 miles of the National Hospital and carters for a mix of population consisting of middle class and low socioeconomic class population from the outskirt of Abuja city. The pediatric facility has 25 beds with 784 annual admissions and 13,716 outpatients. It is staffed by one Attending Pediatrician and four medical officers.

##### f) Maitama Hospital

Maitama Hospital Abuja (MHA) is a government run general hospital within Abuja city. This facility provides care for middle class and low socioeconomic class population and has a large clientele from some of the new periubran settlements on the outskirt of Abuja. It is staffed by one attending pediatrician and four medical officers.

##### g) Federal Medical Center Keffi

This is a general hospital which is owned by the Federal Government and provides clinical services to the semi-urban population of Keffi and several rural settlements. This facility is staffed by one Attending Pediatrician and five medical officers.

The laboratories for this surveillance are primarily based at ZMC. This site was instrumental in supporting the generation of preliminary data that led to the development of this project. The Center for Disease Control and Prevention, Atlanta provided logistic support for setting up the study laboratories and also provided control bacteria strains.

Prior to the commencement of this study, two senior Microbiology Scientists were identified for special training for study procedures and the operation of the Bactec culture instrument. Training was provided at the Medical Research Council Laboratories in the Gambia and upon return to Nigeria, these Technicians have continued to provide supervision and guidance for other laboratory staff. Since commencement, we have procured an additional Bactec™ 9050 culture instrument through a generous donation from Beckton Dickinson, which is now placed at the National Hospital. All the facilities are within 30-45 minutes drive to either of the laboratories.

### Participant enrolment

Following an initial period of sensitization and training for medical officers and residents, blood culture bottles and vacutainer sets were made available to the different sites.

We restricted enrollment initially to just NHA and ZMC and gradually expanded to include the peripheral sites at Gwagwalada, Nyanya, Keffi Medical center and subsequently, GHA and MHA. Not all eligible subjects were approached for enrollment because of insufficient manpower to commit specifically for this study at the different enrollment sites. Enrollment depended very much on the availability of existing medical and nursing staff at the different locations for subject enrollment and also for completion of the questionnaire. Some of the sites are currently understaffed and this clearly retarded our ability to enroll more subjects and to obtain additional specimens such as CSF from clinically suspected cases of meningitis. Such detailed clinical evaluation is currently not routine care at most of these facilities due to lack of adequate diagnostic laboratory services or affordability by caregivers.

Full scale enrolment commenced in November 2008 after formal training was provided to all laboratory personnel. Children who presented with fever or hypothermia were identified by a triage nurse and consent for participation was sought.

Children attending the out-patient clinics or emergency units were triaged and evaluated for eligibility. Informed consent was obtained from the parent or guardian. A physician administered a detailed questionnaire to obtain information on clinical history and physical examination findings. Appropriate clinical specimens were then obtained and these include 1-3 ml of blood for culture using the vacutainer set after cleaning the skin with alcohol swabs. The specimen was collected directly into the Bactec™ culture bottle and promptly delivered by the project driver to ZMC microbiology laboratory within 4 hrs of collection.

### Data Acquisition

The Biomedical Research and Informatics Core (BRIC), Michigan State University provided support and supervision for data management of this study. BRIC is a data management unit for MSU that is involved in nearly 100 active clinical research protocols. Primary research informatics support is provided by a locally developed database application known as *RIX*.

### Metadata design and entry

For the CABSYNC study data was collected from several diverse sources. Forms were entered during the medical exam, at the laboratory after tests specimen processing, and at the time of follow-up. In order to streamline data entry medical examinations forms were acquired from the National Hospital in Abuja (NHA) and laboratory forms from Zankli Medical Center. Next, online forms were designed for RIX to closely mimic the actual forms used onsite. This helped ensure that physicians and laboratory technicians would not have to deviate much from their normal workflow to enter study forms. Data collection instruments that are used for online data capture tools are designed differently as compared to paper forms. CABSYNC forms were edited to reduce rates of implicit uncertain responses.

### Training

The onsite staff in Abuja had to attend an online seminar conducted by the Human Research Protection Program at Michigan State University. This seminar is required for anyone who is given credentials to the RIX system. Additionally, the onsite staff needed to be trained to properly use the RIX system. We used Skype for communicating across borders.

Training sessions included: RIX training for data entry, training for specimen tracking, barcode printing, and printer maintenance and methods of subject tracking and data integrity checks.

### Data Cleaning and analysis

RIX is capable of adding many restrictions to data entry fields but for some variables these fields were left unrestricted. For this pilot stage of data collection it was not possible to predict some of the responses for string fields. During the post pilot data collections phase some of these variables have been coded. Using frequencies of some of the data collected during this pilot phase, it has been possible to provide selectable options for future CABSYNC forms instead of open string fields. Data analysis was performed with Predictive Analytics Software version 19.

### Laboratory Methods

#### Blood culture

In order to determine the etiology of bacteremia, the impact of prior antibiotic exposure on culture yield was minimized by the use of the Bactec™ culture system with culture bottles containing an antibiotic-removing device (antibiotic resins). Bacteria were isolated from blood using an automated blood-culture system (Bactec™ 9050, Becton Dickinson, Temse, Belgium). The study utilized only aerobic blood culture bottles. The work-up for a positive culture vial included a subculture using sheep blood agar or chocolate agar, both of which were supplemented with micronutrients, e.g., iso-vitalex or vitox, for enhanced growth of fastidious bacteria, or McConkey agar plates. Inoculated media was incubated under aerobic and 5% CO_2 _conditions at 35°C for 18-24 hours. Bacteria were identified by a combination of morphology, Gram stain, and chemical methods; for *S. pneumoniae*, by susceptibility to ethyldrocupreine hydrochloride (Optochin) and bile solubility; for *H. influenzae *type b, X and V growth factor dependency detection and slide agglutination with type-specific antisera (bioMerieux, France) and for Enterobacteriacae, we used the API 20 E system (bioMerieux, France). Antibiotic susceptibility assessments were determined by Kirby-Buaer disc diffusion test using standard interpretative criteria and using antibiotic discs for locally available antibiotics for the immediate management of patients.

The blood agar plates for isolating bacteria from blood cultures were either obtained commercially from (Remel) Fisher Scientific USA or when this was not available, sheep blood was obtained from a local abattoir. Sheep blood was collected in to a citrate phosphate donor bag and used for preparation of 5% blood agar plates or chocolate agar plates, using previously described methods for collection and defribrination [15].

Following primary identification of the bacteria isolates at the study site lab, confirmation was sought at reference laboratories. This service was provided by the Medical Research Council Laboratories, The Gambia and Sparrow Hospital Microbiology laboratory, an affiliate of Michigan State University. Michigan State Department of Health Laboratory confirmed the typing of the Salmonella species.

Bacteremia was defined as the isolation of at least one non-contaminant bacteria from the admission blood culture. Coagulase-negative staphylococci, Bacillus, Corynebacterium species or Micrococcoci were classified as contaminants. S. viridians isolates were considered significant when isolated from children with underlying risk factors for S. viridians infection, such as congenital heart defect.

### Antimicrobial activity detection in serum

#### *Micrococcus luteus*-

ATCC 7468 (provided by the Centers for Disease Control and Prevention, Atlanta) was grown overnight on agar plate. Colonies from fresh culture plates were suspended in normal saline, and calibrated to 0.5 McFarland standard. Using another cotton-tipped-sterile applicator, this was inoculated on to a nutrient agar plate, streaking the entire surface of the plate, rotating the plate 60°between streaks and ultimately rimming the plate to ensure confluent growth to the edges. The inoculum was allowed to dry for 5 minutes before depositing the 6-mm sterile paper filter discs. Using a sterile forceps 2 sterile discs were applied ~3 cm apart from each other, taking care not to remove and replace any disc after it has been applied. Once all of the above processes have been accomplished, add 20 μl of the test serum in one disc and 20 μl of sterile saline to the other disc. A positive control disc of chloramphenicol was used in each experiment. The inoculum was allowed to dry and the plates inverted so that the media is on the upper surface and cannot be contaminated by condensation. Plates were then incubated at 37°C in air atmosphere for 18-24 hours before final reading. A ruler was used to measure the zone of inhibition. Any inhibition zone bigger than the saline disc (6 mm) was interpreted as positive for previous antibiotic use.

## Results

From September 2008 to November 2009, children who presented with an acute febrile illness at the enrolling facilities were screened and blood sample collected for bacterial culture. 1,287 children were screened and enrolled, of these 227 children were outside the age range and 91 subjects had incomplete clinical data. These were excluded from further analysis. Thus 969 children aged between 2 months -5 years with a median age of 17 months (+/- 13.9 months) constitute the subject of this pilot report.

Bacteria was isolated from the blood stream of 204 children and of these, 93 were considered skin contaminants and not clinically relevant. Thus significant bacteremia occurred in 111/969 (11.5%) children.

**Figure 1:**
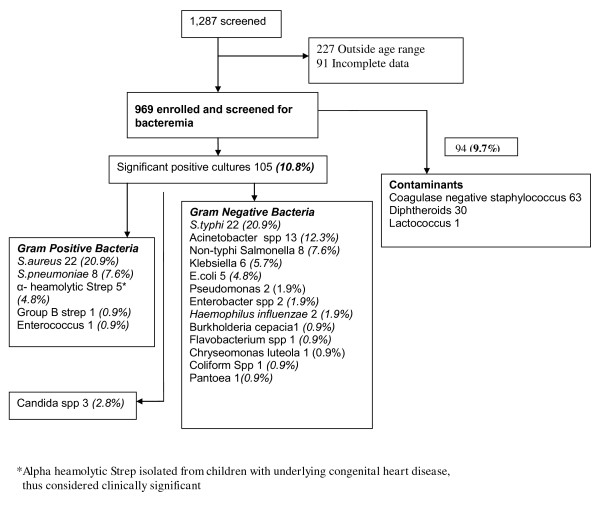
**Summary of Etiologic Agents of Bacteremia in Children aged 2 months-5 years**.

The etiology of bacteremia is summarized in Figure 1. Salmonella spp were the leading cause of bacteremia (28.5%) with *S.typhi *predominating at 20.9% and non-typhi salmonella (7.6%). *S. aureus *accounted for 20.9% while Acinetobacter and *S.pneumoniae *accounted for 12.3% and 7.6% respectively. In five subjects alpha hemolytic streptococcus was isolated and considered clinically significant as these children had underlying congenital cardiac disease.

Concordance between results from the site laboratory and the reference lab was 100% for non-typhi Salmonellae (NTS), *Candida, Escherichia coli *and *Pseudomonas*. The concordance was lowest for alpha hemolytic streptococcus as a number of these were initially mis-identified as *S. pneumoniae*. The identification assigned by the reference laboratory was used for final analysis.

The two *Haemophilus influenzae *isolates were not recovered following primary storage and so we were unable to type these organisms. Of the eight confirmed *S.pneumoniae *isolates, three were 19F; two were 23F and one each of serotypes 1, 5 and 6B.

### Antimicrobial activity in serum

**Figure 2:**
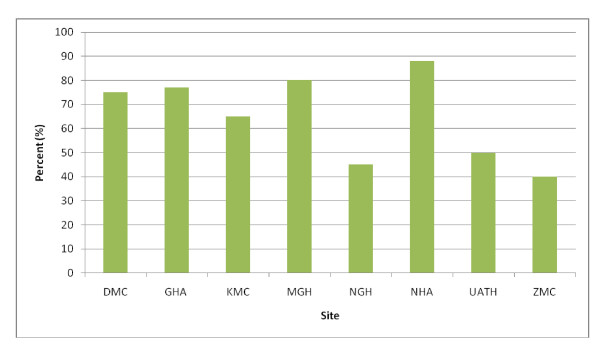
**Antimicrobial Activity in Serum by site of Enrolment**.

Approximately 10% of samples from each enrollment site were randomly selected for testing. Prevalence of serum antimicrobial activity was highest in children who presented to the National Hospital (88.9%), the major tertiary referral center in Abuja and was lowest in those who presented to Zankli Medical Center (40%) and Nyanya General Hospital (45%). Data summarized in Figure 2.

In all, one hundred twenty six sera samples were tested for antimicrobial activity (Table 1). Of these, forty eight tested negative and seventy eight were positive. Of the samples that tested negative, forty six were negative for bacterial growth and two tested positive for bacterial growth (*S. pneumoniae *1 and coagulase negative staphylococcus). Of the samples that tested positive for antimicrobial activity, eight had positive bacterial culture (coagulase negative Staphylococcus 5, *E.coli *1, *S.typhi *-1 and *S. pneumoniae*-1).

**Table 1:** Antimicrobial Activity in Serum and Blood Culture Results

Bacterial Culture Result	Negative Antimicrobial Activity	Positive Antimicrobial Activity
Culture Negative	46	70
**Culture Positive**	CNS*-1 *S. pneumoniae*- 1	*E.coli *- 1 CNS- 5 *S.pneumoniae*- 1 *S.typhi*- 1

CNS*- Coagulase negative Staphylococcus

### Antibiotic susceptibility

A variable number of bacterial isolates were available for testing using different antibiotic discs. All *S. aureus *isolates from the study were screened for methicillin resistance and all were methicillin susceptible. However, susceptibility varied among the spectrum of antibiotics. Of thirteen isolates, all were susceptible to amoxicillin and 20/20 were susceptible to Augmentin with the lowest susceptibility rates seen with cefuroxime 4/12 (33%) and septrin (cotrimoxazole) 12/21 (57%).

Of the *S.typhi *isolates susceptibility was highest (100%) for ceftazidime, ceftriaxone, ciprofloxacin and ofloxacin. Only 10/17 isolates were susceptible to amoxicillin, 9/15 (53%) to augmentin and 11/19 to chloramphenicol. Susceptibility was lowest for septrin 10/21 (48%). *S. pneumoniae *isolates were highly susceptible (100%) to augmentin, amoxycillin, clindamycin and ceftriaxone. However, only three isolates were tested for amoxicillin and clindamycin. *S.pneumoniae *isolates were least susceptible to septrin.

### Clinical Diagnosis and Bacteremia agent

Of the children with a bacteriologic diagnosis of *S.aureus *bacteremia, 4/22 (17%) children presented with clinical sepsis or bronchopneumonia and 2/22 (10%) presented each with clinical diagnosis of febrile convulsion, acute gastroenteritis or pharyngotonsilitis. A large proportion 10/22 (45%) were assigned a clinical diagnosis of malaria. In this setting all children who present with an acute febrile illness are treated empirically for malaria and we did not systematically examine blood smears for malaria parasites. Children with *S. typhi *bacteremia were also most often assigned a clinical diagnosis of malaria 12/21 (55%) and sepsis in only 5/21 (24%) of cases. The clinical diagnosis of bacterial meningitis was made in 2/6 (33%) of children who had pneumococcal bacteremia and in both cases there was CSF pleocytosis but CSF culture was negative. A clinical diagnosis of malaria was made less often in these patients 2/13 (6%).

### Underlying diagnosis

Underlying medical condition was ascertained by clinical history in all participants (Table 2). There were fifty nine children with a known diagnosis of sickle cell disease and thirty two with HIV-infection.

**Table 2:** Distribution of Bacteria Isolate in Children with Established Diagnosis of Sickle Cell Disease or Human Immune Deficiency Virus (HIV) Infection

Bacteria Isolate	Sickle Cell Disease (n = 59)	HIV (n = 32)
*S.aureus*	0	2
*S.pneumoniae*	3	2
*S.typhi*	3	0
*E.coli*	0	1
NTS	2	0
Acinectobacter spp	3	0
Klebsiella	0	1
Pseudomonas	1	0
Contaminant	5	5
No Growth	42	21

Of the children with SCD, 12/59 (20% had bacteremia caused by a clinically significant pathogen). There were three children with each of the following *S.pneumoniae, S.typhi *and Acinetobacter, two children with NTS and one with Pseudomonas spp.

There was no information available on clinical staging or viral load data on HIV-infected children at the time of presentation. Of the HIV-infected children 6/32 had bacteremia caused by a clinically significant pathogen, with *S. aureus *and *S. pneumoniae *in two children and only one child had either *E.coli *or Klebsiella.

### Deaths during hospitalization and phone follow-up

Our data collection system and personnel were inadequate for capturing all deaths following hospitalization. However, we were able to capture deaths which occurred typically less than 72 hrs after subject enrollment, since laboratory culture results were usually made available to the attending physicians within this time frame. Fifteen deaths were recorded during this time frame for a minimum case-fatality ratio of 1.5%. The subject age, sex, initial clinical diagnosis and blood bacterial isolates are listed in Table 3.

**Table 3:** Deaths at Hospital Discharge

ID	Sex	Age in Months	Clinical Diagnosis	Bacterial Isolate
63	F	2	Malaria	NG
87	M	10	SCD, meningitis	*S.pneumoniae*
310	F	4	Malaria	NG
343	M	35	Malaria	Salmonella Spp
351	F	4	Malaria, HIV	NG
436	M	6	Malaria	NG
562	F	5	CHD, Pneumonia	NG
761	F	48	Sepsis, HIV	*H.influenzae*
771	F	8	Malaria	S.typhi
777	M	14	Malaria	NG
790	M	7	Sepsis, CP	*Acinectobacter*
813	M	57	Malaria	NG
817	F	3	Malaria, HIV	NG
973	M	48	Malaria	NG
974	M	48	malaria	NG

NG-No growth

Malaria diagnosis was based on clinical evaluation only

At conclusion of the pilot phase of this study we initiated a phone call to contact all study participant parent/guardian of children who had a positive culture at initial contact (1 mo-1 yr post enrollment). Of the 212 subjects with a positive culture including contaminants, we were able to reach 149 parents, 53 parents were not reachable including 5 who had no phone numbers listed in the entry form. Of the parents that were reachable, we were able to ascertain eight additional deaths that had occurred after initial enrollment in this study (total case-fatality of 2.4%). The subject age, sex, initial clinical diagnosis and blood bacterial isolates are listed in Table 4.

**Table 4:** Additional Deaths Identified at Phone Follow-up (1 month -1 yr after Enrolment)

ID	Sex	Age in Months	Clinical Diagnosis	Bacterial Isolate
43	M	3	Pneumonia	NG
119	F	4	Gastroenteritis	*P.aerugionsa*
435	M	60	Malaria	NG
522	F	48	Pneumonia	*S.pneumoniae*
525	M	14	SBE, CHD	Flavobacterium
598	M	3	Pneumonia	NG
772	M	6	Malaria, HIV	Salmonella Spp
805	F	8	Sepsis, HIV	*S.pneumoniae*

**CHD-Congenital heart disease, SBE-Subacute bacterial endocarditis**

Malaria diagnosis was based on clinical evaluation only

## Discussion

This pilot study has demonstrated the public health significance of etiologic diagnosis of acute febrile illness in a setting where clinical malaria is over-diagnosed, non-prescription antibiotics are prevalent and empiric prescription antibiotic by physicians is the standard clinical practice. This pilot study was sited at Abuja as it is the federal capital city and it is strategically located for access to both urban and rural populations.

The spectrum of agents of bacteremia from this pilot study differs significantly from reports from the Gambia from a similar age group of children [7]. In our cohort, *S. aureus *and S. typhi were the leading cause. While in the Gambia, the pneumococcus is the leading cause of bacteremia and non-typhi salmonella is much more prevalent without any cases of *S. typhi*. The relatively less significant role of pneumococcal bacteremia is likely to be a significant epidemiologic finding secondary to the high incidence of non-prescription antibiotic use. This is most likely, given that all our pneumococcal isolates are penicillin susceptible and the penicillins are the most readily available oral antibiotics in this setting. It is unlikely that we missed clinical isolates, since we utilized a culture system similar to that which was used in other studies in Africa which have reported *S. pneumoniae *as the leading cause of bacteremia in this age group. The same explanation may also hold true for the scarcity of *H. influenzae *from blood cultures. Although *H. influenzae *type b (Hib) vaccine is not included in the National Immunization Program, it is often available in most private hospitals and offered to those who can afford it. However, it is not likely that it would be that widely used to have impacted disease incidence in the wider community. The relative burden of meningitis caused by these two pathogens may be more significant. More intense surveillance for meningitis is planned in the next phase of this surveillance.

In a recent study from Ibadan [16] which utilized conventional culture methods, of 24 cases of definite meningitis 9 were caused by the pneumococcus and 11 by Hib only 4 were caused by *Klebsiella *spp; among pneumonia cases 9 were caused by pneumococcus and 2 by Hib. Interestingly the bacteria isolates from bacteremia cases are similar to those in the current report. So the spectrum of the bacteria recorded in the Abuja project may be related to the prevalent disease spectrum. Also a recent project undertaken just south of Ibadan indicates high pneumococcal carriage rates (90%) in young infants aged < 9 months. (Adetifa I and Adegbola RA- personal communication). This is similar to the carriage rates in The Gambian villages. There is no reason to believe that in a well conducted population study the pneumococcus and Hib may be as important in Nigeria as in The Gambia or Kenya as causes of pneumonia and meningitis.

There may be a distinct difference in disease epidemiology due to the multiple slum settlements on the outskirt of Abuja which is more readily affordable to the general population than Abuja city. Sanitary conditions in these settlements are usually poor or non-existent. The relatively high incidence of *S.typhi *is very concerning and is an important public health issue. Currently, no country in sub Saharan Africa implements routine immunization with typhoid vaccine and reports from several countries suggest that Non-typhi salmonella may be a more important problem than *S.typhi *and there are efforts towards developing vaccines for NTS, while the currently available typhoid vaccines remain unused due to lack of convincing disease burden evidence. Our pilot data suggest that, at least in this setting there may well be a need for implementing routine immunization with typhoid vaccine. It is noteworthy that the current age spectrum included in this study did not include school age children 5-14 yrs, a subgroup that often has a higher incidence of typhoid fever [17]; thus the observation in our report is likely to be an underestimate.

Although *S, pneumoniae *did not feature as the leading cause of bacteremia, it accounts for 13% of all the deaths observed in this pilot study. The study from Ibadan [16] reported over 80% mortality with pneumococcal meningitis in children and only in one study has there been the observation of delayed mortality following invasive pneumococal infection. Whether these delayed deaths are associated with underlying conditions such as HIV, SCD or other immune defects have not been formally investigated. Nigeria is yet to implement routine immunization with the pneumococcal conjugate vaccine. The limited number of invasive pneumococcal isolates that were available from this pilot study suggests that 75% of invasive pneumococcal disease may be preventable with the 7-valent and 100% with the new 13-valent pneumococcal conjugate vaccine.

The rate of antibiotic use, as demonstrated by detection of residual antimicrobial activity in serum, in the community is high in most of the facilities and lowest at ZMC, private hospital. The clientele at this facility are at a higher socioeconomic status than those presenting to the government facilities and are therefore more likely to present early to a private health care provider. This is an interesting observation that warrants further evaluation since on a large scale this may well impact the pattern of etiologic agents identified in children who present to such a facility as opposed to Government establishments. Future surveillance will incorporate enrolment of children at private clinics that are staffed by trained pediatricians.

We have in the course of this pilot study provided increased awareness for physicians and health care providers on the need to carefully consider other diagnosis in children who present with an acute febrile illness, other than malaria and to seek etiologic diagnosis that will properly guide management, particularly, given the increasing reports of the decreasing incidence of malaria in the sub region^18^. We have also provided additional laboratory skills for the identification of bacteria for laboratory technicians and more importantly, we believe providing this diagnostic service will improve the quality of care and improve clinical outcome.

The etiologic agents and their susceptibility patterns observed in this pilot study and other recent studies raises concerns about the appropriateness of the current treatment guidelines recommended by the Integrated Management of Childhood Illness (IMCI). The first line treatment for most conditions with co-trimoxazole or the use of chloramphenicol for severe pneumonia requires revision given the high prevalence of resistance among the commonly isolated bacteria.

In setting up this pilot surveillance, we encountered a number of challenges which are worth mentioning briefly as these will inform the design of larger studies. First, we observed a high contamination rate due largely to the fact that physicians were not conversant with aseptic blood draw techniques in young children and we also had a rapid turnover of house physicians. Second, although motivation of personnel to participate in the study was initially strong, this waned over time as reflected in the decreased enrolment rate at the different sites. Third, a large number of the study questionnaire and patient sampling for cerebrospinal fluid in suspected cases of meningitis was incomplete. There was a general impression amongst the clinical staff that this clinical research project, while beneficial for patient management, increased their workload. These obstacles will be overcome in future studies which will aim to have dedicated salaried personnel for participant enrolment and obtaining relevant clinical specimens. This study has several limitations. Three of the most important include first, our inability to define a population census from where our participants were drawn and therefore unable to define incidence of bacteremia. While this was not the aim of this pilot study, clearly incidence figures would be very useful. However, this approach will be a challenge given the number of healthcare outlets available to the population. Second, we were only able to evaluate the role of underlying conditions such as SCD and HIV only in those who have an established diagnosis. Ideally we should have tested all participants for this condition to determine the role of these conditions in promoting the risk for specific bacteremia. This approach is planned in the next phase of our surveillance. Third, we were unable to provide systematic subject enrollment and this could have introduced some bias in sampling.

## Conclusions

In conclusion, initial findings from the introduction of an automatic blood culture system in Abuja suggest that the spectrum of agents associated with community-acquired bacteremia in young children in central Nigeria may differ from that observed in most other African countries with a predominance of *S.typhi*. This may be due to environmental factors, antibiotic usage, patient selection or technical factors, and epidemiological studies are urgently needed. While this may in part be due to the multiple new settlements and satellite towns around Abuja which in general have poor water supply and sanitary facilities, there is also a high level of non-prescription antibiotic use.

There is a need to intensify surveillance at both rural and urban settlements, expand population surveillance to include school age children and also to determine if this pattern of infection is similar at other geographic locations in the country. A detailed surveillance study for the determination of the incidence of typhoid fever is being planned. Such surveillance activities will be critical for informing the need for introduction of typhoid fever vaccines in the medium term and improvement of infrastructures in the long term. This expanded surveillance should also provide additional data on the disease burden caused by *S. pneumoniae *which appears currently confounded by widespread antibiotic use.

## Competing interests

The authors declare that they have no competing interests.

## Authors' contributions

*Study concept and design*: SO, KB, KI, KO, DS, PA, UE, TA, MO. *Acquisition of data*: KI, UE and CO, AA. *Analysis and interpretation of data*: SO, RA, WK, DI, GO, DO, KB, KI and RA. *Statistical analysis*: SO, KB and K.I. *Drafting of the manuscript*: SO. *Critical revision of the manuscript for important intellectual content*; LL and RA. All authors read and approved the final version of the manuscript.

## Pre-publication history

The pre-publication history for this paper can be accessed here:

http://www.biomedcentral.com/1471-2334/11/137/prepub
